# Access restrictions to forest resources, rather than COVID-19 bans, drive the selection of firewood species for bonfires during *Festas Juninas* in northeastern Brazil

**DOI:** 10.1186/s13002-024-00677-w

**Published:** 2024-04-04

**Authors:** Iara Vitória de Oliveira Araújo, Diego Centeno-Alvarado, Marcelo Alves Ramos

**Affiliations:** 1 Programa de Pós-Graduação em Ciência e Tecnologia Ambiental, Universidade de Pernambuco, Campus Mata Norte, Nazaré da Mata, PE 55800-000 Brazil; 2 Programa de Pós‑Graduação em Etnobiologia e Conservação da Natureza, Departamento de Biologia, Universidade Federal Rural de Pernambuco, Dois Irmãos, Recife, PE 52171‑900 Brazil; 3 Laboratório de Estudos Etnobiológicos, Universidade de Pernambuco, Campus Mata Norte, Nazaré da Mata, PE 55800‑000 Brazil

**Keywords:** Ethnobiology, Ethnobotany, Socio-ecological resilience, Social-ecological systems, Woody species

## Abstract

**Background:**

The complex interplay of social and environmental factors shapes ecosystems, potentially leading to harmony or conflict, highlighting the importance of understanding these dynamics for coexistence. In developing countries, firewood serves as a primary energy source and plays a role in cultural-religious rituals and festivities. However, the specific patterns of woody species used for the latter remain poorly understood, including the impact of access restrictions to resources and local bans on practices. Therefore, our research focuses on examining how access restrictions to forest resources and bonfire bans due to the coronavirus disease 2019 (COVID-19) impact the cultural-religious tradition of bonfire making during *Festas Juninas* (June festivities) in northeastern Brazil.

**Methods:**

Ethnobotanical fieldwork was conducted in two rural populations in northeastern Brazil between 2021 and 2022. Data were collected through semi-structured interviews, observations, and the guided tour technique. The cultural-religious tradition of bonfire making (i.e., richness of native and exotic firewood species, firewood volume, and the number of bonfires related to this practice) was compared between populations (i.e., differing in access restrictions) and years (i.e., differing in COVID-19-related bans) using Mann–Whitney U tests.

**Results:**

Results revealed significant differences in the richness of native (*p* value = 0.001) and exotic (*p* value < 0.001) firewood species for bonfire making due to access restrictions to forest resources. The number of native species used was higher among the population residing in the area with unrestricted access than among those with restricted access, while a greater number of exotic species was used in the population with restricted access. The rest of the variables were not influenced by access restrictions, and no variables were influenced by COVID-19 bans.

**Conclusions:**

Our study demonstrated that access restrictions to forest resources, rather than COVID-19 bans, drive the selection of firewood species for bonfires during *Festas Juninas* in northeastern Brazil. In addition, as populations remain deeply entrenched in cultural-religious practices amid temporary bans imposed by health crises, there is a pressing need for culturally sensitive environmental policies. Fostering socio-ecological resilience demands a comprehensive approach that encompasses not only environmental factors but also cultural dimensions, which wield a pivotal influence on long-term sustainability.

## Background

Ecosystems worldwide are confronted with imminent threats arising from various anthropogenic disturbances, such as deforestation, resource extraction, rapid urbanization, and climate change [1, 2]. These disruptions not only destabilize ecosystem functioning but also pose significant risks to biodiversity conservation. Biodiversity forms the cornerstone of numerous ecosystem services crucial for human survival and well-being [3]. The intricate interplay between well-being, economic activities, and environmental conditions shapes ecosystems [4]. Such interactions occurring between social and ecological systems can either harmonize for mutual benefit or result in conflict, as illustrated by instances where social well-being thrives at the expense of the environment or vice versa [5, 6]. Therefore, understanding these dynamics is crucial for fostering coexistence between the social and environmental spheres [5].

In developing countries, the extraction of resources from woody species plays an essential role in the social well-being and survival of many human populations. These populations depend on woody species as a primary source of energy and for the construction of houses, fences, as well as in the production of crafts and work tools [7–11]. Additionally, woody species play a significant role in rituals and festivities associated with religiosity, strengthening cultural traditions that represent faith and satisfy spirituality and emotional well-being [12, 13]. Scientific data on the patterns of woody species use in cultural-religious manifestations remain scarce [12]. This scarcity is attributed to the tendency of most research to approach the subject superficially, integrating it into broader scientific objectives [13]. Additionally, some authors highlight similarities with other uses, such as the use of firewood for cooking food and heating or for constructing fences to delineate spaces [12, 14]. This confusion has led to the cultural-religious use of firewood being inaccurately categorized in studies as fuel or construction material [12, 14].

In northeastern Brazil, rural populations collect firewood for cultural-religious purposes during the *Festas Juninas* (June festivities) [12, 14–16]. The *Festas Juninas* represent a strong cultural-religious tradition that occurs annually from the beginning of June and lasts until mid-July, aiming to honor the Catholic saints: Saint Anthony, Saint John and Saint Peter [17, 18]. It is estimated that the festivity originated in Ancient Europe as a way to honor the goddess Juno and celebrate the beginning of the harvest [12]. One of the primary features of these festivities is the tradition of bonfire making, in front of the residences of individuals, using firewood sourced from native species in the region [12, 14–16]. During the bonfire burning, people take advantage of the fire to make typical foods of this festivity, such as roasted corn. Despite the significance of this bonfire making during *Festas Juninas* in the northeast region of Brazil, to the best of our knowledge, no studies have investigated the adaptive strategies adopted by human populations to address: (a) access restrictions to forest resources; and (b) bans on bonfire making due to the coronavirus disease 2019 (COVID-19) pandemic. In the former case, studies on various firewood uses, rather than cultural-religious, have shown that restrictions on access and prohibitions on practices involving its utilization have led to the adoption of adaptive strategies to maintain the social reproduction of these practices [17, 19–21]. This includes the gathering of firewood in anthropized areas, increased use of exotic woody species, and the cultivation of native species in environments close to residences, such as backyard gardens [17, 19–21]. In the latter case, bonfire making was banned due to the release of pollutants harmful to human health through smoke, prompted by highly contagious nature of COVID-19 and its severe impact on the respiratory system [22–24].

Thus, we aimed to investigate how local access restrictions to forest resources influence the cultural-religious practice of bonfire making during the *Festas Juninas*. Additionally, we aimed to explore the impact of local bans resulting from the COVID-19 pandemic on this tradition. To do so, we tested the hypothesis that access restrictions to forest resource and bans on bonfire making shape the composition of firewood species (native or exotic), the volume of firewood collected, and the number of bonfires in this cultural-religious practice. We anticipate discovering lower firewood species richness, reduced volume of firewood collected, and fewer bonfires made in human populations living in area with restricted access to forest resources, compared to those living in areas with unrestricted access. Additionally, we anticipate that the local bans implemented during the COVID-19 pandemic will result in lower firewood species richness, decreased volume of firewood collected, and a decrease in the number of bonfires during the year with such bans on bonfire making, compared to the year without such bans.

## Methods

### Study site

**Fig. 1:**
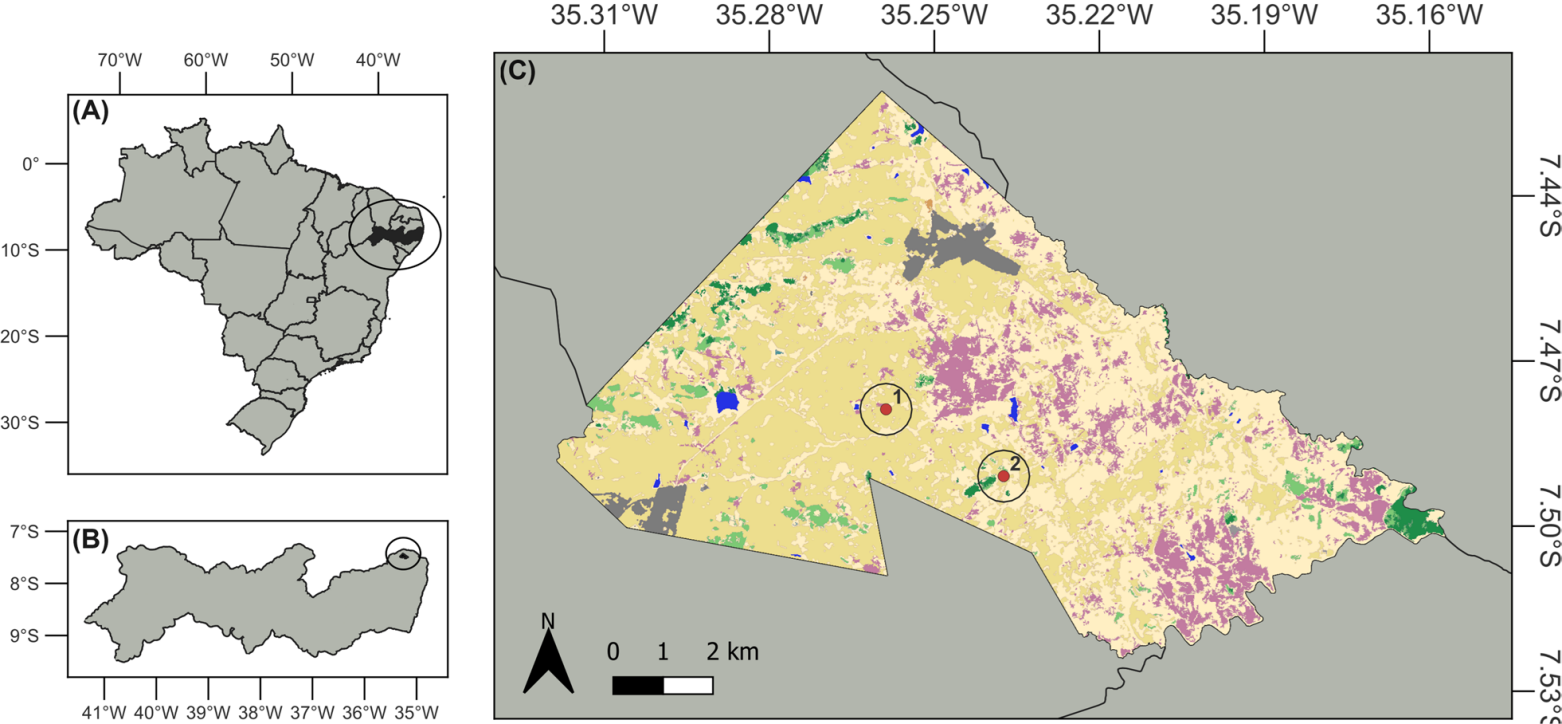
Map of Pernambuco, Brazil, indicated in black (**A**), and the municipality of Ferreiros, also indicated in black (**B**), along with the two human populations studied (**C**) (1: Sítio Cutia; 2: Sítio Barra). Green areas highlight regions with greater coverage of natural forest vegetation. Source of Shapefiles: Brazil and Ferreiros: INPE-National Institute for Space Research, Brazil (public domain); land cover and use: MapBiomas Project, 2022

Our study was conducted in Ferreiros, Pernambuco, northeastern Brazil, situated within the humid tropical forest region known as the Atlantic Forest (7° 26′ 49″ S, 35° 14′ 27″ W). This area is highly fragmented, retaining some remaining fragments of natural forest [25, 26]. The landscape is predominantly characterized by vast sugar cane plantations (*Saccharum officinarum* L.), which constitute the main economic activity in the region. These plantations coexist with subsistence agricultural practices, primarily involving the cultivation of cassava (*Manihot esculenta* Crantz), yam (*Dioscorea cayennensis* Lam.) and sweet potato (*Ipomoea batatas* (L.) Lam.). The municipality covers an area of 88.647 km^2^ and has an estimated population of approximately 12,057 inhabitants, with approximately 19.7% residing in rural areas [27]. The study was conducted in two rural populations, Sítio Barra and Sítio Cutia, which differ in access restrictions to forest resources and are located approximately 10 km apart from each other (Fig. 1).

### Populations studied

#### Sítio Barra

Sítio Barra has a population of 115 inhabitants, distributed across approximately 40 residences. The exclusive ownership of the sole fragment of Atlantic Forest in this region lies with the *Olho D'água* sugarcane processing plant, inherited from its predecessors since the late nineteenth century, around 1889 [28]. As a result, the local population is restricted from accessing these forest resources. Faced with this restriction, the population has been developing several strategies to obtain firewood, including pruning trees in the backyards of their homes and planting native species in agroforestry backyards and less productive agricultural areas.

#### Sítio Cutia

The area of Sítio Cutia is inhabited by a population of 213 people, distributed across approximately 60 residences. Unlike Sítio Barra, the site contains small fragments of Atlantic forest situated on private land owned by local residents. Access to forest resources is unrestricted, both for the landowners and for other individuals residing in the Cutia site, primarily for harvesting firewood for cooking purposes.

### Bans on bonfire making due to the COVID-19 pandemic

Bans on bonfire making due to the COVID-19 pandemic occurred during the sampling period. In 2021, marked by the COVID-19 pandemic and in accordance with the recommendation of the Office of the Attorney General (PGJ) No. 29/2020 [23], the city of Ferreiros, state of Pernambuco, issued Decree No. 28 on June 7, 2021, aligning with state Decree No. 50.778 on June 2, 2021, which established a ban on bonfire making during the *Festas Juninas* throughout the municipal territory. The populations studied were subject to this cultural-religious practice ban. However, on April 22, 2022, the bans were lifted due to the reduction in COVID-19 cases [29]. It is noteworthy that our study specifically evaluates the impact of this ban (i.e., bans implemented in 2021 versus lifted bans in 2022), rather than focusing on periods preceding or during the pandemic.

### Ethnobotanical data collection

Data collection encompassed the *Festas Juninas* of 2021 and 2022 (on the 13th, 23rd and 28th of June), during which we conducted semi-structured interviews with heads of households from both study populations, carried out in Portuguese, their native language (Table 1). In Sítio Cutia, we interviewed 52 out of 61 families (21 women and 30 men), while in Sítio Barra, we interviewed 38 out of 41 families (28 women and 30 men). In addition to the interviews, we adopted observation and measurement of bonfires in each area studied. We also employed the guided tour technique [30] to validate the names of plants mentioned in the interviews. These additional practices provided support for collecting botanical material intended for identification, later incorporated into the herbarium of the Agronomic Institute of Pernambuco. Based on the interviews and observations, we collected:

1. Firewood species used in bonfire making were categorized according to their nature (i.e., native or exotic) and access to forest resources (i.e., restricted or unrestricted), in accordance with the bans on bonfire making in 2021 or 2022.
2. We calculated the number of bonfires per household and the volume of firewood collected in the residences of both areas during the years 2021 and 2022. We used the formula *V* = *w* × *l* × *h*, where *V* denotes the volume of the stack, and *w*, *l*, and *h* represent the width, length, and height of the pile, respectively.

**Table 1:** Form for data collection through semi-structured interviews and measured observations in Sítio Cutia and Sítio Barra during the years 2021 and 2022

*Socioeconomic data about the individual responsible for bonfire making*		
(1)	Full name	–
(2)	Age	–
(3)	Gender	Male Female Other
(4)	Educational level	Not literate or just literate Elementary school I (years 1–5) Elementary school II (years 6–9) High school (years 10–12) Higher education
(5)	Length of residency in the community	*–*
(6)	Occupation of the organizer of the bonfire	–
(7)	Household income	–
*Data on the woody plant species present in the bonfires*		
(8)	Plant name (e.g., common name)	–
(9)	Location of collection	–
(10)	Date of collection	–
(11)	State of collection	Green wood Dry wood
*Bonfire information*		
(12)	Width of the pile (*w*)	–
(13)	Length of the pile (*l*)	–
(14)	Height of the pile (*h*)	–

### Data analyses

We investigated possible differences in the cultural-religious practice of bonfire making (i.e., native and exotic firewood species richness, firewood volume, and number of bonfires) due to access restrictions to forest resources (i.e., Sítio Cutia vs. Sítio Barra) and the bans due to the COVID-19 pandemic (i.e., year 2021 vs. 2022), using the Mann–Whitney U test, with a significance level of 0.05. The statistical analyses were performed using the *stats* package [31] in R [32].

## Results

A total of 234 bonfires were analyzed, with 94 occurring in the population with restricted access to forest resources and 140 in the population with unrestricted access. In the population with restricted access, a total of 27 ethnospecies (Table 2) were utilized for bonfire making. The most frequently observed species were *manga* (*Mangifera indica* L.; 45.74%), *angico* (*Anadenanthera colubrina* var. *cebil* (Griseb.) Altschul; 42.55%), *mermeleiro* (*Croton blanchetianus* Baill.; 41.49%), and *sabiá* (*Mimosa caesalpiniifolia* Benth.; 39.36%). Conversely, in the population with unrestricted access, we documented 44 ethnospecies (Table 2) used for bonfire making. The most prevalent species included *sabiá* (60.71%), *mermeleiro* (42.14%), *angico* (40.00%), *pau d'arco* (*Handroanthus* sp.; 20.00%) and *cajá* (*Spondias mombin* L.; 20.00%).

**Table 2:** Firewood species used for bonfire-making in Sítio Cutia and Sítio Barra during the years 2021 and 2022

Botanical family	Species	Popular name	Status	Frequency (%)		Restrictions due to the COVID-19 pandemic	
				Cutia	Barra	Banned	Lifted
Anacardiaceae	*Mangifera indica* L	Manga	Exotic	16.42	45.74	1	1
	*Spondias mombin* L	Cajá	Native	20	3.19	1	1
	*Astronium urundeuva* (M.Allemão) Engl	Aroeira	Native	2.14	–	0	1
	*Anacardium occidentale* L	Caju	Native	5	–	1	1
	*Spondias dulcis* G. Forst	Cajarana	Exotic	2.14	–	1	1
	*Spondias* cf. *bahiensis*	Cajá-umbu	Native	2.85	–	1	0
Annonaceae	*Annona* sp.	Graviola	Exotic	–	3.19	1	0
Arecaceae	*Cocos nucifera* L	Coco	Exotic	15.71	–	1	1
Bignoniaceae	*Handroanthus* sp.	Pau d’arco	Native	20	23.4	1	1
Bixaceae	*Bixa orellana* L	Açafrão	Native	1.42	–	1	0
Boraginaceae	*Cordia trichotoma* (Vell.) Arráb. ex Steud	Frei-jorge	Native	3.57	–	1	1
Capparaceae	*Cynophalla flexuosa* (L.) J.Presl	Feijão de boi	*Native*	2.14	–	0	1
Combretaceae	*Terminalia catappa* L	Castanhola	Exotic	–	15.95	1	1
	*Combretum leprosum* Mart	Sipaúba	Native	5	–	1	0
Cecropiaceae	*Cecropia palmata* Willd	Imbaúba	Native	–	2.12	0	1
Euphorbiaceae	*Croton blanchetianus* Baill	Mermeleiro	Native	42.14	41.49	1	1
	*Sapium argutum* (Müll.Arg.) Huber	Leiteira	Native	1.42	–	1	0
Lauraceae	*Persea americana* Mill	Abacate	Exotic	0.71	–	0	1
Leguminosae: Caesalpinioideae	*Bauhinia cheilantha* (Bong.) Steud	Mororó	Native	2.85	–	1	1
Leguminosae: Mimosoideae	*Piptadenia retusa* (Jacq.) P.G.Ribeiro, Seigler & Ebinger	Jurema-branca	Native	0.71	–	0	1
	*Anadenanthera colubrina* var. c*ebil (Griseb.) Altschul*	Angico	Native	40	42.55	1	1
	*Enterolobium* sp.	Tambor	Native	2.85	–	1	1
	*Albizia polycephala* (Benth.)	Camudongo / Camuzé	Native	3.57	–	1	1
	*Mimosa caesalpiniifolia* Benth	Sabiá	Native	60.71	39.36	1	1
	*Parkia pendula* (Willd.) Benth. ex Walp	Alucena	Exotic	0.71	6.38	1	1
	*Senegalia tenuifolia* (L.) Britton & Rose	Calombi	Native	3.57	6.38	1	1
	*Prosopis juliflora* (Sw.) DC	Algaroba	Exotic	4.28	–	1	0
	*Samanea saman* (Jacq.) Merr	Bordão de velho	Native	5	–	1	1
	*Caesalpinia pulcherrima* (L.) Sw	Sombrião	Exotic	–	9.57	1	1
Leguminosae: Papilionatae	*Geoffroea spinosa* Jacq	Mari	Native	2.14	–	0	1
	*Machaerium aculeatum* Raddi	Espinho-de-judeu/Espinheiro	Native	12.85	–	1	1
	*Libidibia ferrea* (Mart. ex Tul.) L.P.Queiroz	Jucá	Native	17.14	–	1	1
Malvaceae	*Guazuma ulmifolia* Lam	Mutamba	Native	4.28	–	0	1
Meliaceae	*Azadirachta indica* A.Juss	Nin	Exotic	–	3.19	1	0
Moraceae	*Ficus benjamina* L	Figo	Exotic	5	–	1	1
	*Artocarpus integrifolia* L	Jaca	Exotic	7.44	–	1	1
Myrtaceae	*Psidium guajava* L	Goiaba	Exotic	3.57	10.63	1	1
	*Syzygium cumini* (L.) Skeels	Azeitona	Exotic	1.42	17.02	1	1
	*Eucalyptus globulus* Labill	Eucalipto	Exotic	–	4.25	1	1
Musaceae	*Musa paradisiaca* L	Bananeira	Exotic	–	4.25	1	1
Nyctaginaceae	*Guapira* cf. *noxia* (Netto) Lundell	João-mole	Native	5	–	1	1
Rubiaceae	*Genipa americana* L	Jenipapo	Native	5	–	0	1
Rutaceae	*Citrus aurantium* L	Laranja	Exotic	4.28	20.21	1	1
Sapindaceae	*Talisia esculenta* (Cambess.) Radlk	Pitomba	Native	4.28	–	1	1
	*Allophylus puberulus* (Cambess.) Radlk	Estraladeira	Native	0.71	–	0	1
Poaceae	*Bambusa* sp.	Bambu	Native	2.85	–	1	1
Rhamnaceae	*Ziziphus joazeiro* Mart	Juá	Native	7.14	–	1	1
Vochysiaceae	*Callisthene fasciculata* Mart	Campineiro	Native	2.14	5.31	1	1
Unidentified	–	Canafista	–	3.57	–	1	1
	–	Coração-negro	–	–	1.06	0	1
	–	Piaca	–	0.71	–	1	0
	–	Sete-casca	–	1.42	2.12	0	1
	–	Ameixa	–	2.14	–	0	1

Regarding bans due to COVID-19, 0 represents absence, and 1 represents presence

### Impacts of access restrictions to forest resources and bans on bonfire making due to the COVID-19 pandemic on the cultural-religious practice of bonfire making

**Fig. 2:**
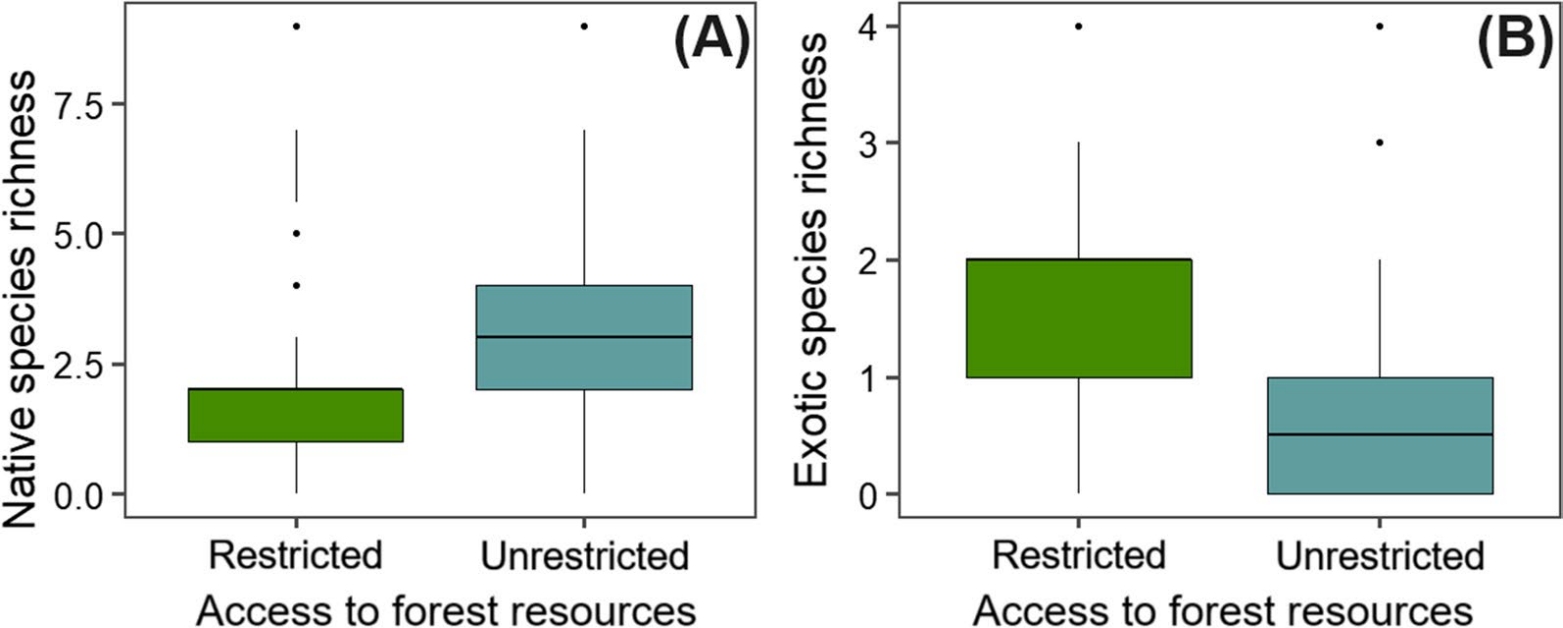
Significant effects of access restrictions to forest resources on the richness of native (**A**) and exotic (**B**) firewood species in the cultural-religious practice of bonfire making in human populations in Ferreiros, Pernambuco, Northeast Brazil. In the graphical representation, the box on the graph represents the interquartile range; the line inside the box represents the median; the whiskers represent the range of non-outlier data; individual points represent outliers

Mann–Whitney U tests revealed significant differences in native and exotic firewood species richness for bonfire making between populations with different access restrictions to forest resources (unrestricted vs. restricted) (Table 3). The number of native species used was higher in the population with unrestricted access than in the population with restricted access (Fig. 2A), while a greater number of exotic species were used by the population residing in the area with restricted access (Fig. 2B). The rest of the variables were not influenced by access restrictions.

**Table 3:** Components of the cultural-religious practice of bonfire making (mean ± standard deviation (SD) and coefficient of variation (CV) (%), through the Mann–Whitney U test

Variable	Access to forest resources		*p* value
	Restricted	Unrestricted	
Native species richness	1.82 ± 1.24; 0.68	2.92 ± 1.98; 0.68	0.001*
Exotic species richness	1.48 ± 1.05; 0.71	0.66 ± 0.82; 1.23	8.08 × 10^–6^*
Firewood volume	1.89 ± 1.26; 0.67	1.69 ± 1.48; 0.87	0.28
Number of bonfires	1.88 ± 0.96; 0.51	1.89 ± 1.08; 0.57	0.80

	Bans due to the COVID-19 pandemic		
	Banned	Lifted	
Native species richness	2.66 ± 1.61; 0.60	2.29 ± 1.97; 0.86	0.16
Exotic species richness	1.06 ± 1.01; 0.95	0.92 ± 1; 1.08	0.38
Firewood volume	1.89 ± 1.22; 0.64	1.66 ± 1.55; 0.93	0.18
Number of bonfires	2 ± 0.91; 0.45	1.77 ± 1.14; 0.64	0.36

*Statistical significance

The analyses also revealed no differences in native and exotic firewood species richness, volume of firewood collected, or the total number of bonfires between the year when bonfire making was banned due to the COVID-19 pandemic (2021) and the year when bans were lifted (2022) (Table 3).

## Discussion

The present study investigated the role of access restrictions to forest resources and the local ban on bonfire making during the COVID-19 pandemic, in the cultural-religious practice of bonfire making during the *Festas Juninas* in two human populations in northeastern Brazil. The results revealed that access restrictions had a significant impact on the investigated cultural-religious practice, influencing the selection of a greater number of exotic species when access to forest resources is restricted, as opposed to a predominance of native species when access is unrestricted. Conversely, the bans on bonfire making during the COVID-19 pandemic did not influence the selection of species, firewood volume collected, or the number of bonfires.

In a study exploring alternative forms of resource usage, such as firewood consumption for cooking, conducted in two protected forest areas (i.e., where access to forest resources is restricted) in Madagascar, it was observed that after the implementation of a ban on firewood extraction, local populations adapted their collection practices [33]. Specifically, they transitioned from preferring specific groups of plants (expert standard) to collecting any dry and available plants without regard to quality (generalist standard). Additionally, the study observed a shift from native to exotic species sourced from anthropogenic environments, along with a decrease in the firewood volume used. The effects of restricted access to forest resources were also observed concerning medicinal and construction use [34]. According to the study [34], restricted access led to changes in disease treatment strategies in the region. People reduced their use of medicinal plants and began relying on biomedical medicines purchased from pharmacies. In terms of construction use, the challenge of accessing mature plants in the forest forced individuals to gather plant species at younger stages from non-forest environments. This directly affected the resistance and quality of the produced pieces. Furthermore, when considering the utilization of wood for crafting musical instruments, it was noted changes in popular culture manifestations due to access restrictions to forest resources [11]. For instance, rather than crafting and using traditional musical instruments, individuals adopted more classical and globalized recognized instruments.

Taken into consideration the previous patterns, the findings of our study indicate the adaptive path followed by the investigated local populations when they are subject to situations of restricted access to forest resources. Cultural adaptations are a common phenomenon for all human groups, occurring when a certain population needs to adjust its knowledge and practices to the limitations imposed by the surrounding environment to maintain a resilient socio-ecological system [35, 36]. Studies in areas with similar prohibitions also highlight cultural adaptation as the main strategy adopted by populations in response to disturbances. [37–40]. In recent years, the concept of resilience has become one of the main conceptual tools in the environmental literature for dealing with change at various levels of organization, from local to global scales [41]. In a resilient socio-ecological system, disruption has the potential to create opportunities for new actions, innovation and development [33, 42].

The finding that bans during the COVID-19 pandemic did not result in significant changes in cultural-religious practices associated with bonfire making indicates a phenomenon of cultural resistance. On the one hand, bonfires have deep cultural and religious significance in the Brazilian northeast region, becoming intrinsically entrenched and challenging practices to be abandoned by the population [43, 44]. On the other hand, the absence of strict supervision, as perceived by residents, encouraged the continuity of bonfire making in 2021, even after the ban, and contributed to the ongoing engagement of people in risky behaviors. This cultural resistance, in the face of prohibitions, highlights the complexity of the interactions between regulatory measures, cultural values, and human behavior. This observation underscores the significant influence of deeply entrenched cultural practices on populations’ responses to temporary bans imposed by health crises.

## Conclusions

Our findings highlight the importance of understanding and incorporating the dynamics of cultural adaptation into management strategies of socio-ecological systems. The ability of human populations to adjust their cultural-religious practices in the face of disturbances, such as access restrictions to forest resources and bans due to the COVID-19 pandemic, points to the need for culturally sensitive environmental policies. Fostering socio-ecological resilience demands a comprehensive approach, that encompasses not only environmental factors but also cultural dimensions, which wield a pivotal influence on long-term sustainability.

## Data Availability

The dataset and R codes are under moderation in Mendeley Data (10.17632/3tprt675dd.1), in order to check that everything is in order and if so they will approve the dataset for publication. We will let you know when this happens (2 business dats from submission, which was today).

## Abbreviations

COVID-19: Coronavirus disease 2019
PGJ: Office of the Attorney General
